# Effects of Single Compared to Dual Task Practice on Learning a Dynamic Balance Task in Young Adults

**DOI:** 10.3389/fpsyg.2018.00311

**Published:** 2018-03-12

**Authors:** Rainer Kiss, Dennis Brueckner, Thomas Muehlbauer

**Affiliations:** ^1^Department of Health and Social Affairs, FHM Bielefeld, University of Applied Sciences, Bielefeld, Germany; ^2^Division of Sports Medicine and Engineering, Hochschule Koblenz, University of Applied Sciences, Remagen, Germany; ^3^Division of Movement and Training Sciences/Biomechanics of Sport, University of Duisburg-Essen, Essen, Germany

**Keywords:** skill acquisition, stabilometer, postural control, cognitive interference task, human

## Abstract

**Background:** In everyday life, people engage in situations involving the concurrent processing of motor (balance) and cognitive tasks (i.e., “dual task situations”) that result in performance declines in at least one of the given tasks. The concurrent practice of both the motor and cognitive task may counteract these performance decrements. The purpose of this study was to examine the effects of single task (ST) compared to dual task (DT) practice on learning a dynamic balance task.

**Methods:** Forty-eight young adults were randomly assigned to either a ST (i.e., motor or cognitive task training only) or a DT (i.e., motor-cognitive training) practice condition. The motor task required participants to stand on a platform and keeping the platform as close to horizontal as possible. In the cognitive task, participants were asked to recite serial subtractions of three. For 2 days, participants of the ST groups practiced the motor or cognitive task only, while the participants of the DT group concurrently performed both. Root-mean-square error (RMSE) for the motor and total number of correct calculations for the cognitive task were computed.

**Results:** During practice, all groups improved their respective balance and/or cognitive task performance. With regard to the assessment of learning on day 3, we found significantly smaller RMSE values for the ST motor (*d* = 1.31) and the DT motor-cognitive (*d* = 0.76) practice group compared to the ST cognitive practice group but not between the ST motor and the DT motor-cognitive practice group under DT test condition. Further, we detected significantly larger total numbers of correct calculations under DT test condition for the ST cognitive (*d* = 2.19) and the DT motor-cognitive (*d* = 1.55) practice group compared to the ST motor practice group but not between the ST cognitive and the DT motor-cognitive practice group.

**Conclusion:** We conclude that ST practice resulted in an effective modulation of the trained domain (i.e., motor or cognitive) while only DT practice resulted in an effective modulation of both domains (i.e., motor and cognitive). Thus, particularly DT practice frees up central resources that were used for an effective modulation of motor and cognitive processing mechanisms.

## Introduction

In everyday life, situations involving the processing of motor (balance) and cognitive tasks simultaneously [i.e., dual task (DT) situations] represent the norm rather than an exception. For example, recalling schedules for an upcoming team meeting while walking toward the meeting room or talking to colleagues on the phone while crossing a busy street is common in our daily routines. Previous studies in healthy young adults investigating DT situations that involved a balance task (e.g., standing or walking) and a cognitive interference task (e.g., serial subtraction of numbers, memorizing words) primarily reported decrements in balance (i.e., increased postural sway, reduced gait speed) and/or in cognitive (i.e., reduced number of correct calculations, increased error rates) task performance. In fact, Chong et al. (2010) proved that the concurrent execution of a serial subtraction task while standing had a significant detrimental impact on balance (i.e., increase in body sway) and on computation (i.e., decrease in speed and accuracy) performance in healthy young adults (mean age: 25 years; *SD*: 3 years). In another study, Beauchet et al. (2005) showed significant performance decrements in DT compared to single task (ST) condition in young adults (mean age: 24 years; *SD*: 3 years), that is slower gait speed and a reduced number of enumerated figures.

Performance decrements during DT situations have previously been explained by limited cognitive capacities (i.e., “central overload”) (Pashler, 1994) and/or cognitive interference when two tasks share the same processing resources (Wickens, 2008). Well-established theories that have widely been used to explain deficits in DT performance are the concepts of a central processing bottleneck (“single channel model”) (Pashler, 1994; Pashler and Johnston, 1998) and the capacity sharing model (Tombu and Jolicoeur, 2003). The single channel model states that cognitive operations are carried out sequentially and a bottleneck arises whenever two tasks require a critical amount of cognitive processing capacity at the same time. On the other hand, the capacity sharing model argues that there is a pool of processing resources or networks that can be distributed between different tasks. Whenever more processing resources are devoted to one task, only limited processing capacity remains for the other networks and tasks and performance deficits in the given tasks arise.

To counteract these decrements in motor-cognitive performance and to improve cognitive as well as motor processing capacities in DT situations, the concurrent practice of both the motor (balance) and the cognitive task may represent a promising approach. Indeed, it has been shown that balance training induces a shift in activation from cortical to subcortical areas (Taube, 2012), indicating an effective modulation of central processing mechanisms (Taube et al., 2007, 2008). In old adults, it is well-documented that DT practice is suitable to improve balance and/or cognitive task performance under DT test conditions (Silsupadol et al., 2009a,b; Hiyamizu et al., 2012; Uemura et al., 2012). However, only a few studies are available in the literature that examined the effects of ST compared to DT practice on balance and cognitive task performance in healthy young adults. For example, Pellecchia (2005) examined the effect of ST versus DT training on balance and cognitive task performance in healthy adults aged 18–46 years. DT training included concurrent practice of the balance (i.e., quiet standing on a compliant surface) and cognitive (i.e., serial three subtractions) task while ST training consisted of practicing the balance and cognitive task separately. Results showed significantly less postural sway in the DT but not in the ST training group when concurrently performing both the balance and cognitive task; yet no significant group differences were detected for cognitive task performance under DT test condition. In another study, Worden and Vallis (2014), investigated the impact of ST compared to DT training on obstacle walking and auditory Stroop task performance in healthy young adults (mean age: 23 years; *SD*: 2 years). DT training included the practice of both tasks simultaneously and ST training consisted of practicing the cognitive task only. They found that only participants in the DT training group significantly improved their walking and Stroop task performance under DT test condition. In summary, studies on the effects of DT practice on balance and cognitive task performance under DT test condition in healthy young adults have shown conflicting evidence (i.e., improvements in both tasks versus improvements in the motor task only). Thus, further research is needed to clarify the impact of ST versus DT training on both balance and cognitive task performance in healthy young adults.

Therefore, the aim of this study was to examine the effects of ST practice (i.e., motor or cognitive task training only) compared to DT practice (i.e., concurrent motor and cognitive task training) on learning a dynamic balance task in healthy young adults. We expected that all three groups would significantly enhance their respective motor (balance) and/or cognitive task performance during 2 days of practice. With regard to the assessment of learning on day 3, we further hypothesized significant group differences during DT test condition in favor of the DT practice group for both motor and cognitive task performance.

## Materials and Methods

### Participants

Forty-eight healthy college student volunteers were randomly assigned to either a ST motor practice group (*n* = 16; eight men, eight women; mean age: 25.0 years; *SD*: 3.1 years), a ST cognitive practice group (*n* = 16; eight men, eight women; mean age: 24.4 years; *SD*: 1.9 years), or a DT motor-cognitive practice group (*n* = 16; eight men, eight women; mean age: 26.1 years; *SD*: 3.4 years). The participants had no prior experience with the experimental tasks and were not aware of the specific purpose of this study. All subjects signed informed consent forms prior to the experiment. The Human Ethics Committee at the University of Duisburg-Essen, Faculty of Educational Sciences approved the study protocol.

### Apparatus and Tasks

#### Dynamic Balance Task

**FIGURE 1:**
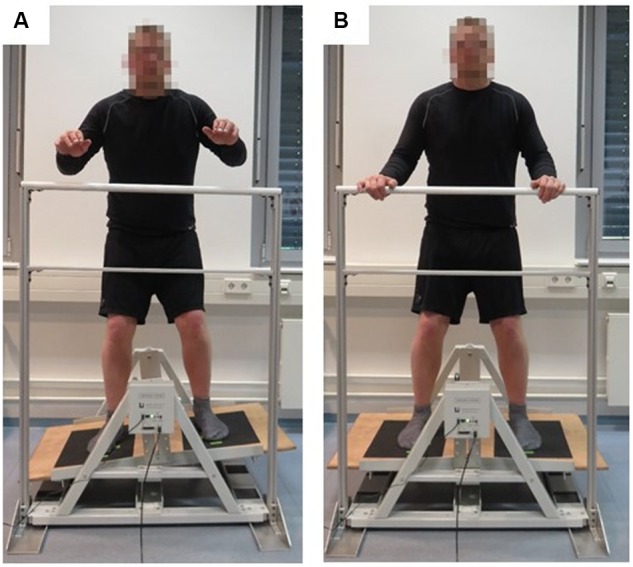
Illustration of a participant balancing **(A)** and standing **(B)** on the stability platform (stabilometer).

The motor task required participants to balance on a stability platform (Lafayette Instrument, Model 16030, Lafayette, CO, United States). The stability platform consists of a 65 × 107-cm wooden platform, allowing a maximum deviation of 15° from the horizontal to either side of the platform (**Figure 1**). A safety rail mounted to the stability platform was used to prevent participants from falling if they lost their balance. Participants were instructed to remain in balance, i.e., to keep the stability platform in a horizontal position for as long as possible during each 90-s trial (**Figure 1A**). A millisecond timer measured time in balance at a sampling rate of 25 Hz. Time in balance was computed when the platform was within ±3° of horizontal position. Additionally, platform position data were exported from the analysis software PsymLab and used to calculate the root-mean-square error (RMSE) in degrees.

#### Serial Subtraction Task

The cognitive task was an arithmetic task, in which the participants loudly recited serial subtractions of three. The subtraction started from a randomly selected number between 300 and 900 that was given by the experimenter (Pellecchia, 2005). If a subject miscalculated, the false calculation was noted. When correctly continuing the serial three subtractions, only one error was noted (i.e., no consequential errors were registered). The total number of subtractions minus the number of subtraction mistakes made during the task was used as outcome measure. Thus, the higher the total number of correct subtractions, the better the cognitive task performance.

### Procedure

In the ST motor and the ST cognitive practice group, participants performed the dynamic balance or cognitive task only, while in the DT motor-cognitive practice group they practiced the dynamic balance task and concurrently performed the cognitive task (i.e., serial three subtractions). All participants were informed that the motor task was to keep the stability platform in the horizontal position for as long as possible during each 90-s trial. Each trial started with the platform in horizontal position and arms grasping the safety rail (**Figure 1B**). Approximately 15 s before the start of a trial, the experimenter asked the participant to step on the platform without shoes. About 3 s before the start of a trial, the experimenter provided the starting number for the serial subtraction task to the participants of the DT motor-cognitive practice group. At the start signal, the participant attempted to move the platform, and data collection began. The arithmetic interference task was chosen because it has previously been shown to mitigate balance performance in healthy young adults (Pellecchia, 2003, 2005; Granacher et al., 2011). All participants performed seven 90-s practice trials on each of two consecutive days of practice under their respective treatment conditions. A 90-s rest interval was given between trials. Knowledge of results (i.e., time in balance and/or total number of correct calculations) was provided after each trial. To assess the learning effects of the different practice conditions, the participants were tested under DT test condition 24 h later (on day 3) without providing knowledge of results.

### Statistical Analyses

During acquisition on day 1 and day 2, the RMSE was analyzed in a 2 (group: ST motor practice, DT motor-cognitive practice) × 2 (day: day 1 to 2) × 7 (trial: trial 1 to 7) analysis of variance (ANOVA) with repeated measures on days and trials. In addition, the total number of correct calculations during acquisition was analyzed in a 2 (group: ST cognitive practice, DT motor-cognitive practice) × 2 (day: day 1 to 2) × 7 (trial: trial 1 to 7) ANOVA with repeated measures on days and trials. During testing on day 3, RMSE and total number of correct calculations while testing under DT condition were analyzed using a one-way ANOVA. Additionally, Cohen’s *d* was calculated to determine whether a statistical difference was practically meaningful as small (0 ≤ *d* ≤ 0.49), medium (0.50 ≤ *d* ≤ 0.79), and large (*d* ≥ 0.80). All analyses were performed using the Statistical Package for Social Sciences (SPSS) version 24.0 and significance level was set at *p* < 0.05.

## Results

### Days 1 and 2: Acquisition

#### Root-Mean-Square Error

**FIGURE 2:**
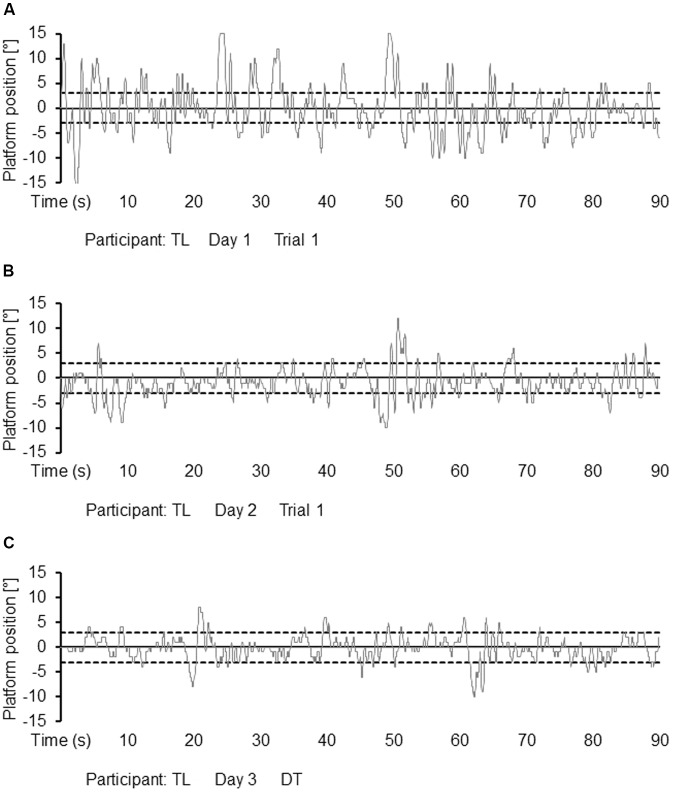
Examples of platform position data profiles for participant TL from the DT motor-cognitive practice group for trial 1 during acquisition on day 1 **(A)**, for trial 1 during acquisition on day 2 **(B)**, and during dual task testing on day 3 **(C)**. DT, dual task.

**FIGURE 3:**
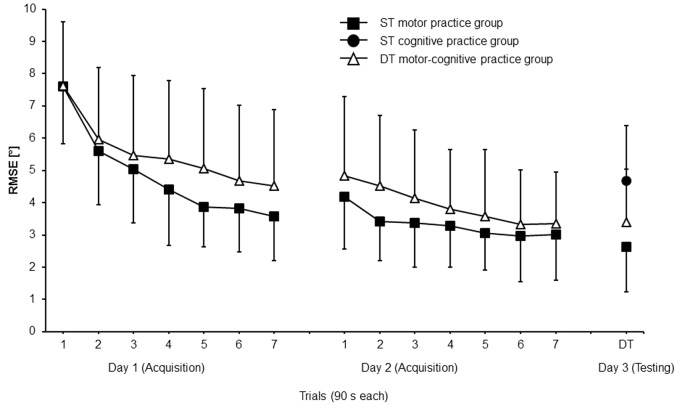
Root-mean-square error (RMSE) of the ST motor and the DT motor-cognitive practice groups during acquisition (day 1 and day 2) and of the ST motor, the ST cognitive, and the DT motor-cognitive practice groups during testing (day 3). Values represent means and standard deviations. ST, single task; DT, dual task.

For a participant from the DT motor-cognitive practice group, examples of platform position data from the first trial on day 1, from the first trial on day 2, and from the DT test condition on day 3 are provided in **Figures 2A–C**. As can be seen from **Figure 3**, both the ST motor and the DT motor-cognitive practice group decreased their RMSE across the 2 days of practice. The Group × Day × Trial ANOVA revealed statistically significant main effects of day, *F*_(1,30)_ = 182.581, *p* < 0.001, *d* = 4.94 and trial, *F*_(6,180)_ = 112.333, *p* < 0.001, *d* = 3.87 but not of group, *F*_(1,30)_ = 1.108, *p* = 0.301, *d* = 0.39. Additionally, we found a significant Group × Day × Trial interaction, *F*_(6,180)_ = 3.713, *p* = 0.002, *d* = 0.70 indicating relatively greater improvements on day 1 than on day 2 in favor of the ST motor practice group.

#### Total Number of Correct Calculations

**FIGURE 4:**
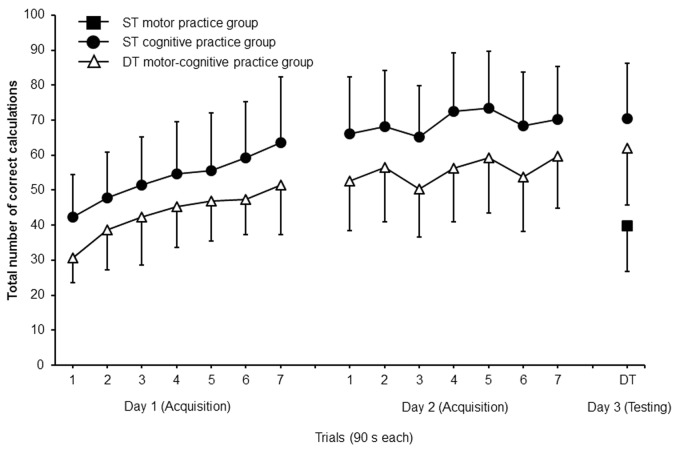
Total number of correct calculations of the ST cognitive and the DT motor-cognitive practice groups during acquisition (day 1 and day 2) and of the ST motor, the ST cognitive, and the DT motor-cognitive practice groups during testing (day 3). Values represent means and standard deviations. ST, single task; DT, dual task.

**Figure 4** displays that both the ST cognitive and the DT motor-cognitive practice group increased their total number of correct calculations over the two practice days. The Group × Day × Trial ANOVA revealed statistically significant main effects of day, *F*_(1,30)_ = 201.406, *p* < 0.001, *d* = 5.17, trial, *F*_(6,180)_ = 50.395, *p* < 0.001, *d* = 2.59, and group, *F*_(1,30)_ = 6.402, *p* = 0.017, *d* = 0.92. The main effect of group indicates a higher level for the total number of correct calculations for the ST cognitive compared to the DT motor-cognitive practice group. The Group × Day × Trial interaction, *F*_(6,180)_ = 1.428, *p* = 0.206, *d* = 0.43 did not reach the level of significance.

### Day 3: Testing

#### Root-Mean-Square Error

The one-way ANOVA showed significant differences between the three groups, *F*_(2,45)_ = 6.759, *p* = 0.003, *d* = 0.48. *Post hoc* comparisons indicated significantly smaller RMSE values under the DT test condition for the ST motor (*p* = 0.002, *d* = 1.31) and the DT motor-cognitive (*p* = 0.040, *d* = 0.76) practice group compared to the ST cognitive practice group. No significant difference was found between the ST motor and the DT motor-cognitive practice group (**Figure 3**).

#### Total Number of Correct Calculations

The one-way ANOVA revealed significant differences between the three groups, *F*_(2,45)_ = 18.730, *p* < 0.001, *d* = 0.61. *Post hoc* comparisons indicated significantly larger total numbers of correct calculations under the DT test condition for the ST cognitive (*p* < 0.001, *d* = 2.19) and the DT motor-cognitive (*p* < 0.001, *d* = 1.55) practice group compared to the ST motor practice group. No significant difference was detected between the ST cognitive and the DT motor-cognitive practice group (**Figure 4**).

## Discussion

In the present study, we compared the effects of DT practice (i.e., concurrent motor and cognitive task training) compared to ST practice (i.e., motor or cognitive task training only) on learning a dynamic balance task in healthy young adults. In accordance with our first hypothesis, we found that all groups significantly improved their respective motor (i.e., decreased RMSE) and/or cognitive (i.e., increased total number of correct calculations) task performance across the 2 days of practice. Contrary to our second hypothesis, we detected similar but not significantly better motor and cognitive task performance for the DT practice group compared to the ST practice groups under DT test condition. However, the ST practice groups improved their performance only in the trained domain. In other words, ST motor practice resulted in enhanced motor but not cognitive performance and the ST cognitive practice lead to better cognitive but not motor performance in DT condition. Only DT practice resulted in improvements in both domains (i.e., enhanced motor and cognitive performance in DT condition).

### Motor (Balance) Task Performance

In contrast to previous research (Pellecchia, 2005; Worden and Vallis, 2014), we found similar but not significantly better balance performance under DT test condition for the DT motor-cognitive practice group compared to the ST motor practice only group. Methodological differences in terms of the used balance task during practice may account for the discrepancies in findings. In this regard, a static balance task (i.e., quiet standing) was used in the study of Pellecchia (2005) and a dynamic balance task (i.e., crossing obstacles while walking) was applied by Worden and Vallis (2014). We also used a dynamic balance task but in contrast to the demands of the walking task used by the latter authors (i.e., stabilizing the center of mass within the base of support during ambulation to adequately perform the task), our dynamic balance task required participants to keep their balance on an unstable but stationary platform and not during ambulation. There is evidence in young adults that static and dynamic components of balance are not related to each other (Muehlbauer et al., 2013). Thus, it can be speculated that different neuromuscular mechanisms are responsible for the regulation of standing (Pellecchia, 2003) and/or walking (Worden and Vallis, 2014) compared to balancing on a stability platform.

A possible ‘ceiling effect’ may additionally account for the non-significant differences between the motor and the motor-cognitive practice group in balance task performance under DT test condition. In other words, the applied training volume (i.e., number of practice trials multiplied by duration per trial) resulted in an overlearning effect (i.e., automatization of the balance task), thus increasing the individuals’ capacity to perform the cognitive task during DT (i.e., mitigating cognitive task interferences). Yet, previous studies that also used the stabilometer device applied the same or a higher training volume to induce practice-related changes in balance performance (Wulf et al., 1998, 2003; Shea and Wulf, 1999; McNevin et al., 2003). Alternatively, the used cognitive interference task was not difficult enough to elicit decrements in balance task performance under DT test condition. However, serial three subtractions were used in former research and resulted in an effective manipulation of attentional demand in healthy young adults indicated by a deteriorated balance performance (Pellecchia, 2003; Granacher et al., 2011; Beurskens et al., 2016).

### Cognitive Task Performance

Contrary to previous research (Worden and Vallis, 2014), we detected similar but not significantly better cognitive task performance for the DT motor-cognitive practice group compared to the ST cognitive practice group in DT test condition. However, only DT practice resulted in an effective modulation of both domains (i.e., motor and cognitive) while ST practice resulted in an effective modulation of the trained domain (i.e., motor or cognitive) only. Thus, our findings on motor and cognitive task performance under DT test condition suggest that particularly DT practice can result in an effective concurrent execution of a serial subtraction and a dynamic balance task. What are likely explanations for this observation? Particularly DT practice seems to be suitable to economize cognitive as well as motor processing capacities during DT situations that are then used to improve arithmetic computation and postural control. Previously, it has been shown that structural and functional changes in the human brain are likely to occur after relatively short periods of practice on the stability platform (i.e., after 2 of 6 practice sessions using 30-s trials) in healthy young adults (Taubert et al., 2010, 2011, 2016). That is, increased gray matter volume in frontal and parietal regions of the brain (Taubert et al., 2010) and increased functional fronto-parietal network connectivity (Taubert et al., 2011). These findings are indicative for an effective modulation of central processing mechanisms following practice of the stabilometer task. Also, the “challenge point framework,” proposed by Guadagnoli and Lee (2004) might be suitable to explain our findings. The authors state that information about the task to be learned and its subjective difficulty is crucial for the learning process. The DT practice situation in our study might have provided the right amount of task difficulty and information to facilitate motor and cognitive learning processes.

Results of the present study revealed significantly improved motor (balance) and/or cognitive task performance across 2 days of acquisition. In addition, we found similar motor and cognitive task performance for the DT practice group compared to the ST practice groups under DT test condition. However, participants in the ST practice groups improved their trained performance only (i.e., motor or cognitive performance) while subjects in the DT motor-cognitive practice group improved both, their motor and their cognitive performance. Our findings are indicative of an effective modulation of the trained domain (i.e., motor or cognitive) only through ST practice but an effective modulation of both domains (i.e., motor and cognitive) through DT practice. Thus, DT practice seems to be suitable to free up central resources that were then used for an effective modulation of motor (postural control) and cognitive (arithmetic computation) processing mechanisms.

### Limitations

Our study includes two limitations that need to be addressed. First, on day 1 we did not assess the initial level of ST and DT performance to compare it with the respective performance on day 3. Consequently, we are not able to adjust our results to a potential difference in ST and DT performance between groups at baseline. Second, we investigated a cohort of healthy young adults whose motor and cognitive capacities are well-developed. Thus, this cohort might be less likely to be influenced by DT situations compared to more impaired cohorts, such as older adults or clinical cohorts. However, even young adults showed impaired motor and/or cognitive performance during DT situations in previous studies (Pellecchia, 2003, 2005; Granacher et al., 2011) and thus might benefit from specific DT practice protocols. Although, a generalization of our results to other (clinical) cohorts is not advisable.

## Author Contributions

RK developed the research design and was the primary author of the manuscript. DB collected the data and gave edits throughout the creation of the manuscript. TM helped to create the research design and provided content and edits to the manuscript.

## Conflict of Interest Statement

The authors declare that the research was conducted in the absence of any commercial or financial relationships that could be construed as a potential conflict of interest.
